# m6A epitranscriptomic modification in hepatocellular carcinoma: implications for the tumor microenvironment and immunotherapy

**DOI:** 10.3389/fimmu.2025.1538658

**Published:** 2025-02-17

**Authors:** Fen Liu, Qingbin Liu, Xianying Li, Yufei Wang, Ruoyu Cao, Shiyu Zhang, Shulong Jiang, Jianlin Wu

**Affiliations:** ^1^ Institute of Traditional Chinese Medicine, Shandong University of Traditional Chinese Medicine, Jinan, China; ^2^ Clinical Medical Laboratory Center, Jining First People’s Hospital, Shandong First Medical University, Jining, China; ^3^ College of First Clinical Medicine, Shandong University of Traditional Chinese Medicine, Jinan, China

**Keywords:** hepatocellular carcinoma, tumor immune microenvironment, m6A modification, immune checkpoint inhibitors, immunotherapy resistance

## Abstract

Hepatocellular carcinoma (HCC) is the most prevalent primary liver malignancy and a leading cause of cancer-related deaths globally. The asymptomatic progression of early-stage HCC often results in diagnosis at advanced stages, significantly limiting therapeutic options and worsening prognosis. Immunotherapy, with immune checkpoint inhibitors (ICIs) at the forefront, has revolutionized HCC treatment. Nevertheless, tumor heterogeneity, immune evasion, and the presence of immunosuppressive components within the tumor immune microenvironment (TIME) continue to compromise its efficacy. Furthermore, resistance or non-responsiveness to ICIs in some patients underscores the urgent need to unravel the complexities of the TIME and to design innovative strategies that enhance immunotherapeutic outcomes. Emerging evidence has revealed the pivotal role of N6-methyladenosine (m6A), a prominent RNA methylation modification, in shaping the TIME in HCC. By regulating RNA stability and translation, m6A influences immune-related factors, including cytokines and immune checkpoint molecules. This modification governs PD-L1 expression, facilitating immune escape and contributing to resistance against ICIs. Advances in this field have also identified m6A-related regulators as promising biomarkers for predicting immunotherapy response and as potential therapeutic targets for optimizing treatment efficacy. This review examines the regulatory mechanisms of m6A modification within the TIME of HCC, with a focus on its impact on immune cells and cytokine dynamics. It also explores the therapeutic potential of targeting m6A pathways to improve immunotherapy efficacy and outlines emerging directions for future research. These insights aim to provide a foundation for developing novel strategies to overcome immune resistance and advance HCC treatment.

## 1 Introduction

Hepatocellular carcinoma (HCC) is the most common type of primary liver cancer globally and a leading cause of cancer-related deaths. Its high incidence is often linked to chronic hepatitis virus infections (HBV or HCV), liver cirrhosis, excessive alcohol consumption, and non-alcoholic fatty liver disease (1). Due to the lack of early symptoms, many HCC patients are diagnosed at advanced stages, leading to limited treatment options and poor prognosis. Recently, immunotherapy, particularly immune checkpoint inhibitors, has brought new hope for HCC treatment (2). However, challenges like liver cancer heterogeneity, immune evasion, and immunosuppressive cells in the tumor microenvironment (TME) undermine immune responses (3). Additionally, some patients show resistance or no response, limiting its effectiveness (4).Thus, a deeper understanding of the HCC immune microenvironment and immune evasion mechanisms is crucial for improving immunotherapy outcomes.

Post-transcriptional modifications (PTMs) are critical regulatory processes that occur after RNA is transcribed from DNA. These modifications are widely found in various types of RNA, including messenger RNA (mRNA), transfer RNA (tRNA), ribosomal RNA (rRNA), and non-coding RNA. PTMs play a critical role in regulating gene expression by modulating RNA stability, splicing, translation, transport, and degradation (5). Key types of RNA modifications include N6-methyladenosine (m6A), 5-methylcytosine (m5C), pseudouridine (Ψ), N1-methyladenosine (m1A), and adenosine-to-inosine (A-to-I) editing (6). Among these, m6A is the most well-known and extensively studied, predominantly found in mRNA. m6A is dynamically regulated by specific enzymes: “writers” (e.g., METTL3), “erasers” (e.g., FTO and ALKBH5), and “readers” (e.g., YTHDF1) (7). This dynamic modification allows RNA to quickly respond to internal and external signals, influencing its fate and function.

In recent years, m6A modification has emerged as a key regulator in the immunotherapy of HCC, attracting growing attention. By modulating the expression of immune checkpoint molecules, such as PD-1 and PD-L1, m6A facilitates tumor immune evasion, thereby compromising the anti-tumor activity of T cells (8). Moreover, m6A influences the polarization of tumor-associated macrophages (TAMs), reshaping immune responses within the TME (9). Given the dynamic and reversible nature of m6A, targeting this modification presents a promising therapeutic strategy for HCC immunotherapy (10, 11). Such interventions could enhance treatment sensitivity and foster the development of novel therapeutics. This review aims to elucidate the pivotal role of m6A modification in HCC immunotherapy, dissect its underlying mechanisms in regulating tumor immune evasion and remodeling of the TME, and assess its potential as a therapeutic target for improved clinical outcomes.

## 2 The dynamic regulation of of m6A modification and its role in HCC

### 2.1 The dynamic regulation of m6A modification in cellular processes

**Figure 1:**
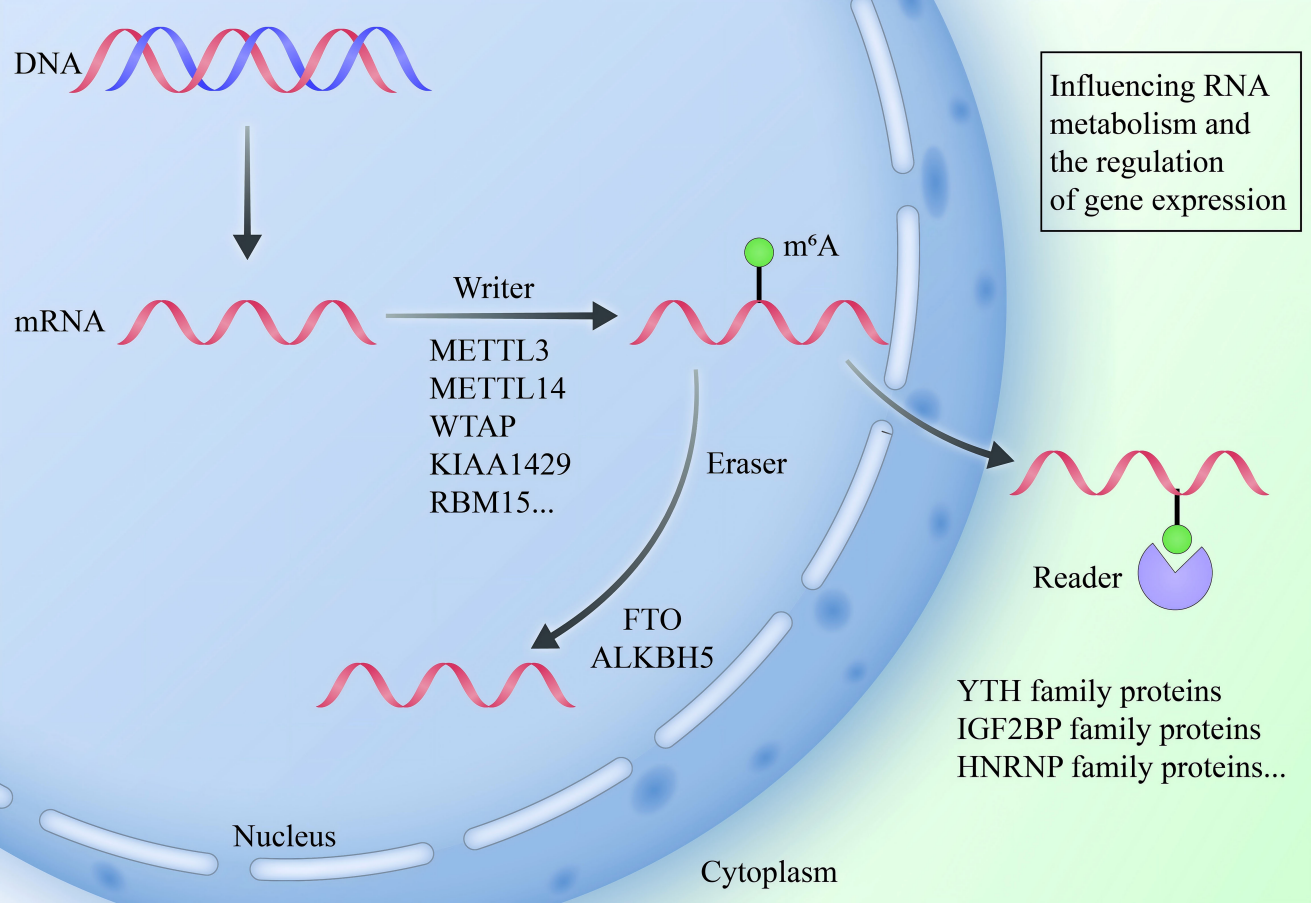
The dynamicregulation of m6A modification. The regulation of m6A modification is mediated by three distinct classes of proteins that collaboratively maintain its dynamic and reversible nature. “Writers,” including METTL3 and METTL14, catalyze the addition of m6A marks, while “erasers,” represented by FTO and ALKBH5, remove these modifications. “Readers,” encompassing the YTH and IGF2BP protein families, decode m6A marks to influence RNA processes, including stability, splicing, and translation. Collectively, these regulators establish a sophisticated epitranscriptomic network that fine-tunes RNA metabolism and gene expression.

The regulation of m6A modification is dynamically controlled by three distinct classes of proteins: “writers,” “erasers,” and “readers.” Acting in concert, these proteins mediate the deposition, removal, and interpretation of m6A marks, ensuring the reversible and precise nature of this epitranscriptomic modification. Collectively, they establish a complex regulatory network that governs RNA metabolism and gene expression, underpinning a wide array of critical cellular processes (**Figure 1**).

#### 2.1.1 Writers

The installation of m6A marks is driven by the m6A methyltransferase complex, with METTL3 and METTL14 functioning as the core catalytic components responsible for methylating the N6 position of adenosine at specific RNA sites (12, 13). METTL3 accounts for catalyzing approximately 95% of m6A modifications on mRNA (14). Supporting proteins such as WTAP, KIAA1429, and RBM15 contribute to site specificity and enhance methylation efficiency (15, 16). This writer complex typically modifies regions near the 5’ and 3’ untranslated regions (UTRs), regulating key processes like RNA stability, splicing, and translation efficiency (17).

#### 2.1.2 Erasers

m6A modification is dynamic and reversible, with its removal facilitated by demethylases, or “erasers.” The primary enzymes responsible for this demethylation are FTO (fat mass and obesity-associated protein) and ALKBH5 (18). FTO was the first demethylase identified (19), with a crucial role in reversing m6A modifications, while ALKBH5 contributes to post-transcriptional regulation of RNA, influencing RNA stability, splicing, and translation (20, 21). By removing m6A, these erasers restore RNA to its unmodified state, affecting its stability and translational capacity.

#### 2.1.3 Readers

The biological effects of m6A modification are mediated by “reader” proteins, which recognize and bind to m6A-modified sites, thereby dictating the fate of the RNA. Major reader proteins include the YTHDF1/2/3 and YTHDC1/2 families, which regulate RNA degradation, transport, splicing, and translation (22, 23). For example, YTHDF1 enhances protein synthesis by promoting mRNA translation, whereas YTHDF2 directs the degradation of m6A-modified RNA. In addition, the IGF2BP family stabilizes m6A-marked mRNAs, further refining gene expression at the post-transcriptional level (24, 25).

### 2.2 Functional roles of m6A in HCC

As a dynamic and reversible RNA methylation process, m6A exerts a profound influence on HCC progression initiation and progression by modulating RNA stability, splicing, translation, and degradation. This biochemical modification governs the expression of both oncogenes and tumor suppressor genes, thereby affecting HCC cell proliferation and apoptosis. The writer enzyme METTL3 facilitates tumor growth by stabilizing oncogene mRNA, whereas erasers such as FTO and ALKBH5 suppress tumor progression by removing m6A marks (26–29). Additionally, m6A enhances HCC cell migration and invasion by regulating metastasis-related genes, including VEGFA and ZEB1, and promoting epithelial-mesenchymal transition (EMT), thus elevating metastatic potential (30, 31). m6A modification also sustains HCC cell stem-like properties by modulating stemness-associated genes, such as *SOX2* and *KLF4*, which contribute to drug resistance and recurrence (32, 33). Furthermore, m6A facilitates immune evasion by modulating immune checkpoint molecules like PD-L1, helping HCC cells escape immune surveillance (34, 35). Given the dynamic and reversible nature of m6A, targeting m6A regulatory enzymes (e.g., METTL3 and FTO) presents a promising therapeutic approach. Modulating m6A modification offers an opportunity to influence HCC proliferation, metastasis, and immune evasion, creating new avenues for HCC treatment.

## 3 Impact of m6A modification on the immune microenvironment in HCC

The TME of HCC possesses unique immunosuppressive characteristics that support tumor growth and enable immune evasion. This TME includes regulatory T cells (Tregs), myeloid-derived suppressor cells (MDSCs), and M2-polarized tumor-associated macrophages (TAMs), all of which release immunosuppressive factors like IL-10 and TGF-β to inhibit anti-tumor responses (3, 36–38). Tumor cells further promote immune escape by expressing PD-L1, which interacts with PD-1 on T cells, reducing T-cell-mediated anti-tumor activity. Hypoxia within the TME intensifies immunosuppression and drives tumor resistance, while the liver’s immune-tolerant nature provides an ideal environment for tumor cell survival and spread (39, 40). Thus, the TME, though challenging, presents a promising target for immunotherapy in HCC.

### 3.1 Regulation of immune cell function by m6A in HCC

#### 3.1.1 T Cells

In HCC, high expression levels of m6A writers, particularly METTL3, are associated with poor prognosis, as they enhance PD-L1 levels and subsequently inhibit T cell-mediated anti-tumor responses (41, 42). METTL3-mediated m6A modification also upregulates the non-coding RNA TUG1, which increases the expression of PD-L1 and CD47 via miR-141 and miR-340 sponging and interaction with YBX1, a transcriptional regulator. This regulation suppresses CD8+ T cell activation, aiding immune evasion and contributing to tumor progression (42). Another recent study found that METTL3 stabilizes SMPDL3A via m6A modification, promoting HCC growth and immune evasion by interacting with IGF2BP1. Knockdown of *METTL3* activated CD8+ T cells, increasing TNFα/IFN-γ production and reducing HCC cell survival. SMPDL3A overexpression reverses these effects (43). Additionally, METTL3 facilitates immune escape in non-alcoholic fatty liver disease-associated HCC (NAFLD-HCC) by promoting cholesterol biosynthesis, which further impairs CD8+ T cell function. Targeting METTL3 in conjunction with PD-1 blockade has demonstrated synergistic effects in restoring CD8+ T cell cytotoxicity (44). WTAP, another m6A writer, stabilizes PD-L1 mRNA, which promotes immune evasion and enhances aerobic glycolysis in HCC cells. This effect suppresses the tumor-killing function of CD8+ T cells, but can be reversed through *WTAP* knockdown, thus improving T cell anti-tumor activity (45). Similarly, the m6A “eraser” FTO stabilizes GPNMB mRNA, which inhibits CD8+ T cell activation through SDC4 receptor binding in small extracellular vesicles (sEVs). Knocking down *FTO* enhances CD8+ T cell recruitment and bolsters anti-tumor responses (28). BMI1, independent of its classical role, influences CD127+KLRG1+ memory CD8+ T cell differentiation in HCC by regulating TCF1 expression. BMI1 interacts with YTHDF2 to prevent m6A-driven degradation of TCF1 mRNA. This regulation shifts T cells towards memory and effector states, enhancing their tumor-killing abilities. Meanwhile, tumor cell-intrinsic BMI1 expression downregulates BMI1 in T cells. Liver-specific *BMI1* knockdown effectively restores CD8+ T cell functionality and supports immunotherapy efficacy in HCC (46). In NASH-HCC, YTHDF1, an m6A reader, promotes MDSC accumulation and suppresses CD8+ T cell functionality via IL-6 secretion. By binding to m6A-modified EZH2 mRNA, YTHDF1 enhances IL-6 production. siRNA targeting of YTHDF1 has shown potential in enhancing anti-PD-1 therapy, making YTHDF1 a promising target in immune-based HCC therapies (47). Exosomal circCCAR1, stabilized by WTAP-mediated m6A modification, promotes HCC growth by forming a feedback loop with miR-127-5p and WTAP. When absorbed by CD8+ T cells, circCCAR1 induces dysfunction by stabilizing PD-1 and promoting resistance to anti-PD1 therapy. This effect is further enhanced through PD-L1 transcription activation by EP300-induced CCAR1 and β-catenin interaction, which reinforces immune evasion (48).

#### 3.1.2 Tumor-associated macrophages

Macrophages in the HCC TME are primarily polarized to the M2 phenotype, which secretes immunosuppressive cytokines like IL-10 and TGF-β, promoting tumor growth and suppressing anti-tumor T cell activity. METTL3- and METTL16-mediated m6A modification stabilizes ZNNT1 in HCC, which enhances macrophage recruitment and polarization to M2 via the osteopontin (OPN)/S100A9 feedback loop, ultimately supporting immune evasion and tumor progression (49). Similarly, the lncRNA miR4458HG, through the m6A reader IGF2BP2, stabilizes key glycolytic mRNAs like HK2 and GLUT1, promoting glucose metabolism and tumor-associated macrophage polarization in HCC, thereby contributing to immune suppression (50). In contrast, circFUT8 downregulation via M1 macrophage-derived exosomal miR-628-5p limits METTL14-mediated m6A modification, reducing circFUT8’s influence on the circFUT8/miR-552-3p/CHMP4B pathway, and suppressing HCC progression by inhibiting tumor growth and immune suppression (51). Additionally, ALKBH5 overexpression in HCC enhances tumor progression by upregulating MAP3K8 through m6A modification, leading to JNK and ERK pathway activation. This triggers IL-8 expression, attracting macrophages and amplifying immune evasion (52). In HBV-related HCC, the METTL3-mediated upregulation of lncRNA MAAS in M2 macrophages drives tumor progression. HBV-associated antigen HBeAg elevates MAAS, which is then transferred to HCC cells through exosomes. Inside HCC cells, MAAS stabilizes c-Myc, promoting cell cycle progression and proliferation, thus highlighting an m6A-regulated feedback loop contributing to HCC malignancy and immune escape (53). Together, these findings underscore m6A modification’s regulatory role in macrophage polarization in HCC, providing multiple potential targets for therapeutic intervention.

#### 3.1.3 Dendritic cells

m6A modification significantly impacts the antigen-presenting function of dendritic cells (DCs), with downstream effects on T cell activation and antitumor immunity. The m6A reader YTHDF1 specifically modulates DC function by influencing neoantigen presentation. In YTHDF1-deficient mice, increased cross-presentation of tumor antigens by DCs strengthens CD8+ T cell activation and antitumor responses. Mechanistically, YTHDF1 binds m6A-modified transcripts of lysosomal proteases, enhancing cathepsin translation and thus limiting cross-presentation. Inhibiting cathepsins in wild-type DCs similarly boosts antigen presentation, demonstrating YTHDF1’s role as a potential immunotherapy target, especially in conjunction with PD-L1 checkpoint inhibitors (54). Under radiotherapy, YTHDF1 upregulation in DCs reduces IFN-I production via STING degradation, impairing DC cross-priming and diminishing T cell activation. Loss of YTHDF1 not only amplifies radiotherapy efficacy in melanoma models but also induces long-lasting immunity, marked by robust CD4+ and CD8+ T cell responses and IFN-γ secretion, as seen in gastric cancer studies (55, 56). Collectively, these findings position YTHDF1 and m6A modulation in DCs as promising targets to enhance T cell-mediated antitumor immunity and improve cancer immunotherapies.

#### 3.1.4 Myeloid-derived suppressor cells

MDSCs, known for their immunosuppressive function, contribute to T-cell inhibition and facilitate tumor immune evasion. Elevated m6A levels enhance the immunosuppressive nature of MDSCs, promoting their accumulation and activation within the TME. In colorectal cancer (CRC), high m6A levels and reduced ALKBH5 expression in MDSCs correlate with increased immunosuppressive capacity. ALKBH5 overexpression reduces m6A levels on Arg-1 mRNA, destabilizing its expression and diminishing MDSC-mediated suppression (57). METTL3 also plays a key role in MDSC regulation. In CRC models, METTL3 promotes MDSC migration by activating the m6A-BHLHE41-CXCL1 pathway. Silencing METTL3 reduces MDSC accumulation and promotes CD4+ and CD8+ T-cell proliferation, suppressing CRC growth. Mechanistic studies reveal that METTL3 induces BHLHE41 expression in an m6A-dependent manner, which in turn activates CXCL1, guiding MDSC migration through the CXCR2 axis (58). In ovarian cancer, Mettl3 deficiency within myeloid cells shifts immune balance from M1 to M2 macrophage polarization, enhancing pro-tumorigenic inflammation via cytokines including IL-1β, CCL2, and CXCL2 (59). This modulation underscores Mettl3’s role in maintaining immune response homeostasis within the TME.

**Figure 2:**
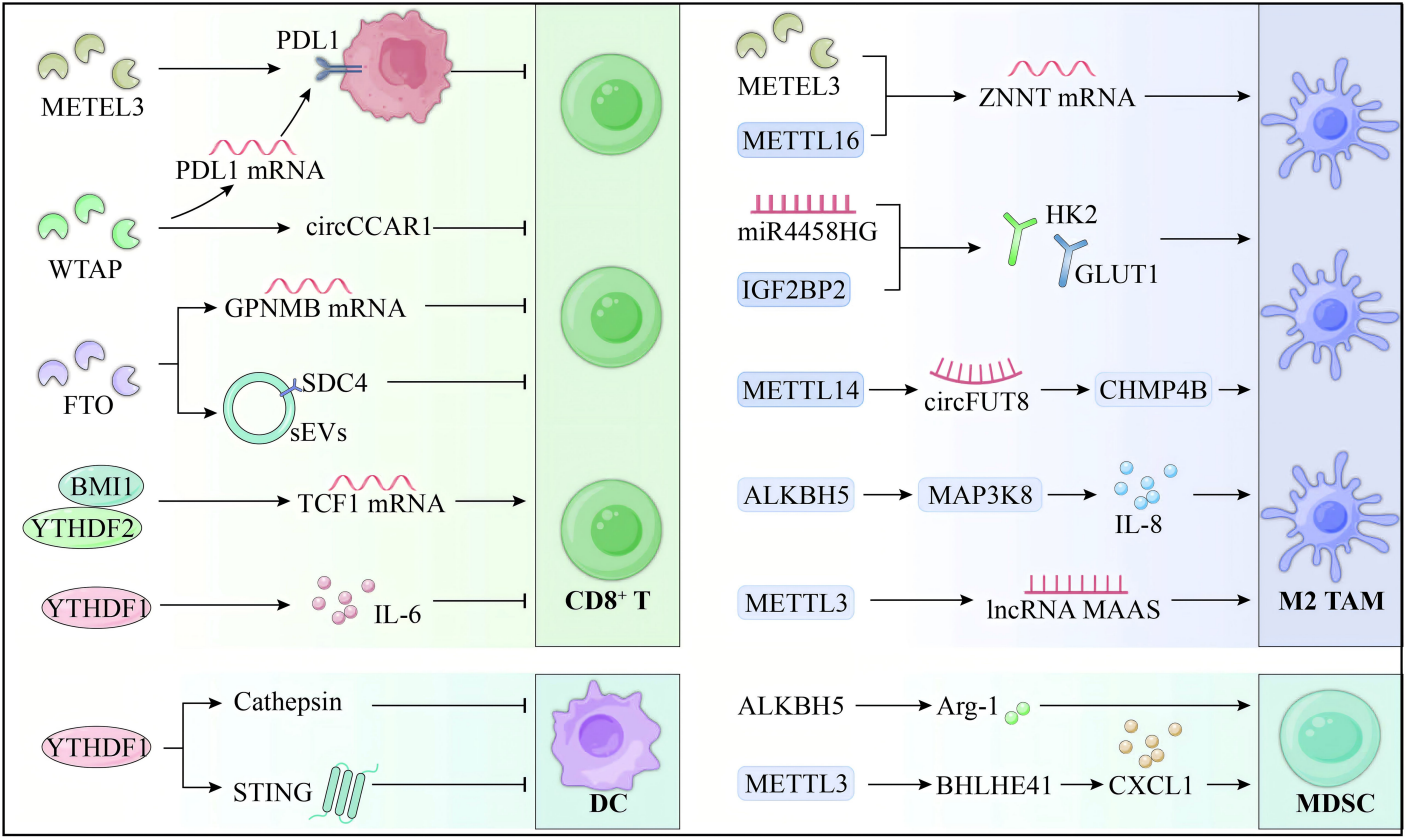
m6A regulation of immune cells in HCC microenvironment. m6A modification plays a critical role in modulating the TIME of HCC, driving immune evasion and affecting therapeutic responses. In T cells, METTL3 promotes PD-L1 expression and suppresses CD8+ T cell activity, while FTO stabilizes GPNMB, further impairing their cytotoxicity. In TAMs, m6A drives M2 polarization through METTL3 and ALKBH5, enhancing immunosuppressive cytokine production. In DCs, YTHDF1 limits antigen presentation, reducing CD8+ T cell activation. Additionally, m6A enhances MDSC immunosuppressive functions. The role of m6A in regulating immune cells within the HCC TIME underscores its potential as a promising therapeutic target to improve immunotherapy outcomes.

**Figure 2** illustrates the m6A-mediated regulation of immune cells within the HCC microenvironment. Although m6A modification is a pivotal regulator of immune evasion and tumor progression in HCC, significant gaps hinder its therapeutic translation. Current research overly focuses on PD-1/PD-L1 blockade in T cells, neglecting alternative immune checkpoints and downstream pathways. The role of m6A-modified non-coding RNAs in immune dysfunction also demands further investigation to identify novel targets. In macrophages, the mechanisms driving M2 polarization and their interaction with immune networks remain poorly understood, and the feedback roles of METTL3 and ALKBH5 within the TME lack clarity. Advancing m6A targeted immunotherapy necessitates a broader investigation into immune interactions, alternative pathways, and HCC specific mechanisms.

### 3.2 Modulation of cytokine dynamics by m6A in HCC

#### 3.2.1 Inflammatory cytokines

##### 3.2.1.1 m6A drives interleukins production

m6A modification plays a crucial role in modulating interleukin (IL) signaling, significantly influencing HCC progression and inflammation. One key interleukin, IL-6, is highly regulated by m6A modification. Enhanced m6A methylation stabilizes IL-6 mRNA, increasing its expression in the HCC microenvironment, which promotes tumor cell proliferation and survival. A notable feedback loop involving exosomal SLC16A1-AS1 stabilizes SLC16A1 mRNA via m6A, enhancing lactate influx and activating the c-Raf/ERK pathway. This pathway induces M2 macrophage polarization, which in turn secretes IL-6, activating STAT3 signaling. STAT3 activation leads to METTL3 upregulation, further stabilizing SLC16A1-AS1 through m6A modification, promoting HCC growth, invasion, and glycolysis (60). YTHDF1 overexpression in NASH-HCC promotes IL-6 secretion through m6A-modified EZH2 (47), resulting in MDSC recruitment and CD8+ T-cell suppression. Similarified GNAS upregulation by LPS stimulation enhances IL-6 expression by elevating m6A methylation of mRNA, while *GNAS* knockdown diminishes IL-6, thereby inhibiting tumor growth (61).

Beyond IL-6, reduced YTHDF2 in HCC increases m6A-modified IL-11 mRNA, exacerbating inflammation and metastasis by impairing the degradation of IL11 and SERPINE2 mRNAs (62). Additionally, upregulated m6A-modified RNA AC026356.1 enhances cancer progression by binding IGF2BP1, stabilizing IL11 mRNA, and activating IL11/STAT3 signaling (63). In chronic liver inflammation, MeRIP-seq analysis revealed reduced m6A methylation of IL-17RA mRNA, linked to inflammation-driven HCC. The demethylase FTO, rather than METTL3, primarily mediates this reduced methylation (64). Furthermore, in alcoholic steatohepatitis (ASH), chronic alcohol intake induces Kupffer cell pyroptosis and increases IL-1β release. Silencing METTL3 alleviates this inflammation by regulating pri-miR-34A splicing (65), highlighting METTL3 as a key modulator of interleukin-driven inflammation and a potential therapeutic target.

##### 3.2.1.2 m6A amplifies TGF-β effects

m6A modification has emerged as a pivotal regulator of TGF-β signaling, profoundly influencing cancer progression and immune modulation. In HCC, TGF-β-induced METTL3-mediated m6A modification destabilizes ITIH1 mRNA, disrupting fibronectin and focal adhesion kinase signaling, thus driving tumor growth and invasion (66). Additionally, TGF-β induces m6A modification that destabilizes PCDHGA9 mRNA, facilitating HCC progression and metastasis (67). In gastric carcinoma, aberrant overexpression of the m6A regulator WTAP stabilizes TGF-β mRNA, promoting cell migration, EMT, and resistance to chemoradiotherapy (68). Similarly, in breast cancer, YTHDC1 enhances the stability of m6A-modified SMAD3 mRNA, potentiating TGF-β signaling and enabling lung metastasis. Loss of YTHDC1 disrupts EMT and cell migration, highlighting its critical role in TGF-β-mediated tumor progression (69).

In addition to malignancies, m6A modification interconnects metabolic and inflammatory pathways in liver diseases. In NASH, LPS-induced NF-κB activation upregulates METTL3/METTL14, driving hypermethylation of TGF-β1 mRNA at its 5’ UTR, enhancing translation and linking inflammation to fibrosis (70). Correspondingly, the liver progenitor-specific gene *RALYL* mitigates m6A modification on TGF-β2 mRNA, stabilizing its expression and activating the PI3K/AKT and STAT3 pathways to promote tumorigenicity and metastasis in HCC (71).

##### 3.2.1.3 Interferon modulation by m6A

As key anti-tumor cytokines, the expression of interferons is also regulated by m6A modification. In radiation-induced liver diseases (RILD), irradiation triggers ALKBH5 to demethylate m6A residues in the 3’ UTR of HMGB1, activating the STING-IRF3 pathway and promoting the production of type I interferon (IFN), which contributes to hepatocyte apoptosis. Loss of ALKBH5 or silencing of HMGB1 reduces IFN levels and inflammation. Also, YTHDF2 facilitates the degradation of m6A-modified HMGB1, further linking m6A to the regulation of liver inflammation and apoptosis (72). In liver cancer, MATR3 inhibits IFN signaling by binding to DHX58 mRNA, recruiting YTHDF2, and promoting mRNA degradation. MATR3 knockout restores IFN signaling, enhancing CD8+ T cell-mediated tumor elimination (73). In HBV-related HCC, elevated serum pgRNA levels are associated with poor prognosis. IFN-α-2a enhances m6A modification of pgRNA, leading to its destabilization and reduced tumorigenicity, suggesting that targeting m6A-modified pgRNA may improve IFN signaling and offer a potential therapeutic strategy for HCC (74).

#### 3.2.2 Chemokines

Multiple studies have established that m6A RNA modification regulates chemokine expression by modulating mRNA stability and translation. In acute liver failure (ALF), tristetraprolin (TTP) upregulation enhances m6A modification of CCL2 and CCL5, leading to mRNA destabilization and reduced expression. This m6A-mediated degradation, driven by enzymes such as METTL14, alleviates liver injury (75). In intrahepatic cholangiocarcinoma (ICC), hepatocyte-secreted CCL3 promotes metastasis via VIRMA-mediated m6A modification, which upregulates SIRT1 and drives tumor progression (76). Similarly, hepatitis B virus (HBV) surface antigens (SHBs) increase CCR9 expression through KIAA1429-mediated m6A modification, stabilizing CCR9 mRNA and facilitating HCC progression and regorafenib resistance (77). This stabilization positions CCR9 as a critical therapeutic target and prognostic biomarker in HBV-related HCC. Additionally, the m6A reader protein YTHDF2 stabilizes Cx3cl1 mRNA in peritumoral hepatocytes, enhancing CD8+ T cell recruitment and activation via the cGAS-STING pathway (78). The m6A-dependent process bolsters immune responses, improves immunotherapy efficacy, and suppresses liver tumor growth. In metabolic-associated fatty liver disease (MAFLD), reduced METTL14 expression diminishes GLS2 translation via the m6A/YTHDF1 axis, exacerbating oxidative stress and recruiting pro-fibrotic Cx3cr1+ macrophages. These macrophages activate hepatic stellate cells via the CX3CR1/MyD88/NF-κB signaling axis, contributing to liver fibrosis (79). Restoring METTL14 expression or inhibiting MyD88 signaling alleviates fibrosis, offering promising avenues for intervention in MAFLD.

**Figure 3:**
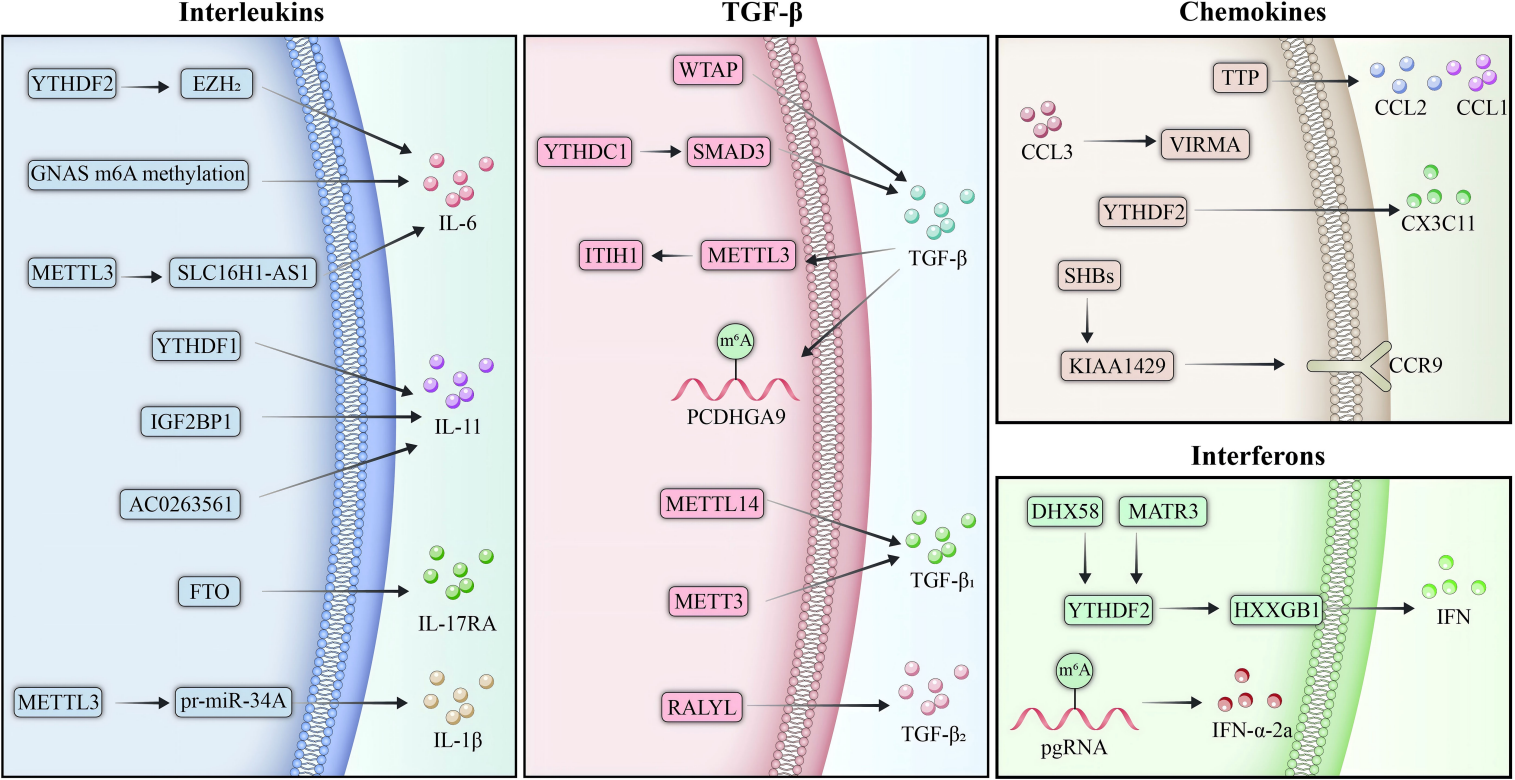
m6A regulation of inflammatory and chemokine signaling in HCC. m6A modification serves as a pivotal regulator of inflammatory cytokines and chemokines in the TME of HCC, shaping immune responses and driving tumor progression. Stabilization of IL-6 mRNA by m6A enhances STAT3 activation, promoting immune suppression through M2 macrophage polarization and recruitment of MDSCs. Similarly, reduced YTHDF2 expression leads to the stabilization of m6A-modified IL-11 mRNA, further amplifying inflammation and metastasis. TGF-β signaling is tightly modulated by m6A, where METTL3-mediated destabilization of tumor-suppressive transcripts facilitates tumor growth and links inflammation to fibrosis. In addition to cytokines, m6A regulates chemokine system, with KIAA1429 stabilizing CCR9 mRNA in HBV-related HCC, driving metastasis and resistance to therapy. Conversely, YTHDF2-stabilized Cx3cl1 promotes CD8+ T cell recruitment and activation, underscoring m6A’s dual role in HCC.

m6A modification critically regulates inflammatory cytokines and chemokines, shaping HCC progression and immune evasion (**Figure 3**). Stabilization of IL-6 mRNA by m6A promotes tumor proliferation, while aberrant m6A modifications of IL-11 and IL-17RA exacerbate inflammation and metastasis. TGF-β signaling and interferon pathways, modulated by m6A, further drive invasion, immune suppression, and resistance. Chemokines such as CCL2 and CCL3 are similarly affected, altering the TME and therapy responses. However, the current understanding is limited by the lack of precise, *in vivo* models to dissect m6A’s temporal and spatial effects. Future research should integrate multi-omics, single-cell technologies, and m6A targeted therapeutics to refine mechanistic insights and clinical applications.

## 4 Potential applications of m6A modification in HCC immunotherapy

ICIs, including PD-1/PD-L1 inhibitors (e.g., nivolumab and pembrolizumab) and CTLA-4 inhibitors (e.g., ipilimumab), have emerged as a central immunotherapy strategy for liver cancer, acting by restoring T-cell activity through the blockade of immune checkpoint molecules. Despite their promise, patient response rates to ICIs remain suboptimal, with resistance being a frequent challenge. Recent studies highlight the pivotal role of m6A modification in modulating PD-L1 expression and immune cell function within the TME. Targeting m6A may attenuate tumor immune evasion mechanisms, thereby enhancing the anti-tumor efficacy of ICIs in liver cancer.

### 4.1 Influence of m6A regulation on the efficacy of immune checkpoint inhibitors

The m6A methyltransferases METTL3 and METTL14 intricately orchestrate immune responses by selectively modulating specific mRNA targets. METTL3 amplifies the translation of SCAP mRNA in NAFLD-HCC, promoting cholesterol biosynthesis while simultaneously impairing the cytotoxic capacity of CD8+ T cells. Inhibition of METTL3 synergizes with anti-PD-1 therapy, thereby restoring immune functionality (79). Similarly, METTL14 facilitates the degradation of Siah2 mRNA through m6A modification and YTHDF2-mediated decay in cholangiocarcinoma (CCA). Siah2 promotes PD-L1 ubiquitination, enhancing immune evasion. Suppressing Siah2 significantly improves the therapeutic efficacy of immune checkpoint inhibitors (ICIs) (80). In intrahepatic cholangiocarcinoma (ICC), the m6A demethylase ALKBH5 has been shown to diminish m6A methylation on PD-L1 mRNA, shielding it from YTHDF2-mediated degradation. This stabilization sustains PD-L1 expression, suppressing T-cell activation and reshaping the tumor immune microenvironment (TIME) to favor immune escape. Notably, tumors with elevated nuclear ALKBH5 expression exhibit heightened sensitivity to anti-PD-1 therapy (81).

The m6A readers YTHDF1 and YTHDF2 play distinct but complementary roles in modulating immune responses. YTHDF1 enhances the translation of EZH2 mRNA in NASH-HCC, which promotes IL-6 secretion and the recruitment of MDSCs, collectively impairing CD8+ T-cell function (47). Strikingly, YTHDF1 silencing, when combined with anti-PD-1 therapy, significantly suppresses tumor progression. Moreover, circRHBDD1 functions as a cofactor for YTHDF1, directing it to PIK3R1 mRNA, thereby driving aerobic glycolysis and contributing to resistance against anti-PD-1 therapy (82). Conversely, YTHDF2 is essential for maintaining mitochondrial fitness and chromatin remodeling in CD8+ T cells via m6A-dependent RNA decay, processes critical for sustaining T-cell polyfunctionality (83). Loss of YTHDF2 diminishes the efficacy of ICIs, whereas inhibition of IKZF1/3 restores T-cell functionality, underscoring its therapeutic relevance.

Circular RNAs (circRNAs) stabilized by m6A modifications have also been identified as critical mediators of immune evasion and therapeutic resistance. In HCC, circCCAR1, stabilized through WTAP-mediated m6A modification, forms a feedback loop with miR-127-5p, upregulating WTAP expression. Secreted via exosomes, circCCAR1 stabilizes PD-1 expression in CD8+ T cells, inducing dysfunction and resistance to ICIs (48). Similarly, circRHBDD1 facilitates immune escape by recruiting YTHDF1 to PIK3R1 mRNA (82), thereby enhancing glycolysis—a vulnerability that can be therapeutically targeted to improve the efficacy of PD-1 blockade. One recent study revealed that TAM transfer m6A-modified circPETH via extracellular vesicles to HCC cells, where it encodes circPETH-147aa. This protein promotes glycolysis and metastasis by facilitating PKM2-catalyzed ALDOA-S36 phosphorylation and impairs CD8+ T cell function by stabilizing SLC43A2 mRNA, driving resistance to immune checkpoint blocker (ICB). Norathyriol, a small molecule targeting circPETH-147aa, reverses these effects, enhancing anti-PD1 therapy and restoring CD8+ T-cell activity, highlighting its potential in overcoming ICB resistance in HCC (84). Another study demonstrated lncRNA TUG1, upregulated by METTL3-mediated m6A modification, drives HCC immune evasion. The combination of TUG1-siRNA therapy with anti-PD-L1 antibodies exhibits synergistic tumor suppression effects (42).

Additionally, m6A modifies IDO1 expression through the IFN-γ/JAK1/STAT1 pathway, promoting immune escape and PD-L1 upregulation. IDO1 inhibition with Abrine reduces PD-L1 expression, enhances macrophage phagocytosis, and, when combined with anti-PD-1 therapy, improves T cell responses, suppressing tumor growth (85).

The intricate interplay between m6A regulatory pathways and immune responses unveils numerous therapeutic opportunities (**Table 1**). Targeting m6A writers (METTL3/14), erasers (ALKBH5), readers (YTHDF1/2), and circRNA-driven pathways (circCCAR1, circRHBDD1) represents a promising strategy to overcome resistance to ICIs.

**Table 1:** Roles of m6A regulators in immune responses and therapeutic potential.

Key Molecules	Mechanism	Impact	Therapeutic Strategy	Ref.
METTL3	Enhances the translation of SCAP mRNA in NAFLD-HCC, promoting cholesterol biosynthesis; impairs cytotoxicity of CD8+ T cells.	Promotes immune evasion and tumor progression.	METTL3 inhibitors combined with anti-PD-1 therapy restore immune function.	(79)
METTL14	Facilitates degradation of Siah2 mRNA via m6A modification and YTHDF2-mediated decay.	Siah2 degradation reduces PD-L1 ubiquitination, enhancing immune evasion.	Suppressing Siah2 enhances the efficacy of ICIs.	(80)
ALKBH5	Reduces m6A methylation on PD-L1 mRNA, shielding it from YTHDF2-mediated degradation, maintaining high PD-L1 expression.	Inhibits T-cell activation, reshapes the TIME, and enables immune escape.	Tumors with high nuclear ALKBH5 expression exhibit sensitivity to anti-PD-1 therapy.	(81)
YTHDF1	Enhances EZH2 mRNA translation, promoting IL-6 secretion and MDSC recruitment, impairing CD8+ T-cell function; drives PIK3R1 mRNA aerobic glycolysis via circRHBDD1, causing resistance to anti-PD-1 therapy.	Promotes immune suppression and therapeutic resistance.	Silencing YTHDF1 combined with anti-PD-1 therapy significantly inhibits tumor progression.	(47) (82)
YTHDF2	Maintains mitochondrial fitness and chromatin remodeling in CD8+ T cells via m6A-dependent RNA decay, sustaining T-cell polyfunctionality.	Loss of YTHDF2 diminishes ICIs efficacy; IKZF1/3 inhibition restores T-cell functionality.	A key molecule in maintaining T-cell function with therapeutic target potential.	(83)
circCCAR1	Stabilized by WTAP-mediated m6A modification; forms a feedback loop with miR-127-5p to upregulate WTAP; secreted via exosomes, stabilizes PD-1 in CD8+ T cells, inducing dysfunction and resistance to ICIs.	Enhances immune evasion and resistance to anti-PD-1 therapy.	Targeting circCCAR1 and its associated pathways could enhance anti-PD-1 therapy efficacy.	(48)
CircPETH	m6A-driven circPETH-147aa enhances PKM2 activity and SLC43A2 stability, promoting HCC glycolysis, metastasis, and CD8+ T cell dysfunction.	circPETH-147aa attenuats CD8+ T cell mediated immunity against HCC.	Norathyriol, an inhibitor of circPETH-147aa, enhances anti-PD1 efficacy.	(84)
Circ RHBDD1	Recruits YTHDF1 to PIK3R1 mRNA, enhancing glycolysis and promoting immune evasion.	A key driver of resistance to anti-PD-1 therapy.	Inhibiting circRHBDD1 could improve the efficacy of anti-PD-1 therapy.	(82)
LncRNA TUG1	TUG1 upregulated by METTL3 modified m6A sponges miR-141/miR-340 and binds YBX1 to elevate PD-L1/CD47.	TUG1 shows a positive correlation with PD-L1 and CD47.	TUG1-siRNA and anti-PD-L1 synergistically suppress HCC.	(42)

### 4.2 Potential of m6A as a biomarker in immunotherapy

m6A modification levels show promise as potential biomarkers for predicting HCC patient responses to immunotherapy. Integrating m6A-related gene expression with immunotherapy regimens could provide a basis for personalized treatment, improving patients’ responses to immunotherapy. Machine learning (ML) is a powerful tool for predicting immunotherapy efficacy in HCC, combining multi-omics data such as genomics, immune checkpoint expression, and TIME features. The inclusion of m6A-related gene signatures improves the accuracy of these ML models, facilitating better patient stratification based on tumor mutation burden (TMB), immune infiltration, and m6A modification patterns. The integration of m6A data with clinical and radiomic features offers a more comprehensive approach to personalized immunotherapy, providing insights into the likelihood of response to treatments like anti-PD-1 and CTLA-4 inhibitors. Further research is needed to validate these models for clinical use. **Table 2** summarizes recent m6A-related risk models associated with predicting immunotherapy efficacy.

**Table 2:** m6A-related risk model for predicting immunotherapy efficacy.

m6A-related risk model	Signature	Prediction of immunotherapy	Ref.
m6A-related genes	*IGFBP3*, *TCP1*, *CFHR5*, *HDAC2*, *INTS8*, *UQCRH*, *PABPC4*, *GYS1*, *MARCKSL1*, *MAPRE1*, *GYS2*, *NAP1L1*, *XPNPEP1*, *STX6*, *BLMH*, *YBX1*, *RDH16*, *HDDC2*, *MASP2*, *HMGN1*	High-risk group benefits from immunotherapy.	(86)
m6A regulators genes	9 harmful regulators (*IGF2BP3, IGF2BP2, METTL4*, *HNRNPC*, *HNRNPA1*, *YTHDF1*, *IGF2BP1*, *HNRNPG*, *METTL16* and 4 beneficial regulators (*FMR1*, *METTL14*, *ZC3H13*, *YTHDC2*)	Low m6A.ES.harm subgroup shows better PD-1/CTLA4 response. High m6A.ES.benefit subgroup responds well to CTLA4 blocker.	(87)
m6A-related miRNAs	let-7b-5p, miR-148a-3p, miR-17-5p, miR-182-5p, miR-212-3p, miR-22-3p, miR-652-3p, miR-9-5p, miR-99b-3p	Elevated PD-1/PD-L1 expression links to higher risk score.	(88)
m6A-related lncRNAs	GABPB1-AS1, AC025580.1, LINC01358, AC026356.1, AC009005.1, HCG15, and AC026368.1	High-risk group responds better.	(89)
m6A-associated snRNAs	RNU1-70P, RNU1-75P, RNU6-2, RNU6-94P, RNU11, RNU6-247P, RNU6-1011, RNU6-510P	High-risk group benefits more from immunotherapy.	(90)
m1A-, m5C-, m6A-, m7G-, and DNA methylation-related regulators	BMT2, NEIL3, TRMT6, WDR4, and ZC3H13	Low-risk group shows sensitivity to immunotherapy.	(91)
Combined hypoxia and m6A/m5C/m1A regulated genes	*CSTF2*, *NUP93*, *MAP4*, *RUVBL1*, *CEP55*, *DPH2*, *DNAJC5*, *SMS*, *GNPDA1*, *ATG5*, *GFL1*, *GPD1L*, *ATP1B3*	High-risk group shows better immunotherapy response.	(92)
m6A/m5C/m1A-related genes	*METTL3*, *YTHDF1*, *NSUN4*, *and TRMT6*	High-risk group overexpresses HAVCR2, PDCD1, CTLA4, CD274, and TIGIT.	(93)
m6A/m5C/m1A regulator genes	*YTHDF1*, *YBX1*, *TRMT10C and TRMT61A*	High-risk group exhibits elevated expression of PD-L1, PD-1, CTLA4, HAVCR2, PDCD1LG2, and TIGIT.	(94)
m6A regulators	“readers”: ELAVL1, FMR1, HNRNPA2B1, HNRNPC, IGF2BP1, IGF2BP2, IGF2BP3, LRPPRC, YTHDC1, YTHDC2, YTHDF1, YTHDF2, and YTHDF3; “writers”: CBLL1, KIAA1429, METTL14, METTL3, RBM15, RBM15B, WTAP, and ZC3H13; and “erasers”: ALKBH5 and FTO	Low-m6A score subtype indicates immunosuppression, limited immunotherapy benefits.	(95)
m6A Modification-Related Genes	*YTHDF2*, *YTHDF1*, *METTL3*, *KIAA1429*, and *ZC3H13*	Lower risk score predicts better response and survival outcomes of anti-PD-1 immunotherapy.	(96)
m6A methyltransferase-related lncRNA	LINC01093, LINC02362, SNHG20, SNHG17, ZFAS1, SNHG6, SNHG7 and GAS5	Low risk predicts better immunotherapy response.	(97)
SNRPC expression	SNRPC	Low-SNRPC group responds better to immunotherapy.	(98)

## 5 Discussion and conclusion

m6A modification, a prevalent form of RNA methylation, is emerging as a critical regulator of HCC progression and immune response. Acting as an epigenetic mechanism, m6A influences various cellular processes, including immune cell function, inflammatory cytokine production, and immune checkpoint regulation, ultimately shaping the TIME and impacting the efficacy of immunotherapy. Understanding how m6A alters the immune landscape in HCC reveals its potential as a therapeutic target, particularly for enhancing immunotherapy outcomes.

The TIME within HCC is composed of various immune cells, including T cells, macrophages, and dendritic cells, all of which interact with tumor cells to drive both tumor progression and immune evasion. m6A modification plays a central role in modulating the mRNA stability and translation of molecules that mediate these interactions. For instance, METTL3, a key m6A methyltransferase, promotes the expression of IL-6 (99), a cytokine known to recruit myeloid-derived suppressor cells (MDSCs) and inhibit CD8+ T cell cytotoxicity. This contributes to the suppression of antitumor immunity and supports tumor survival and growth. The m6A reader protein YTHDF1 further exacerbates this effect by enhancing the translation of EZH2 mRNA, which in turn amplifies IL-6 secretion and impairs T cell function (47). These findings suggest that m6A regulators, such as METTL3 and YTHDF1, represent potential therapeutic targets that could help restore immune surveillance and enhance the immune system’s ability to fight tumors.

In addition to cytokine regulation, m6A also influences immune checkpoint expression, which is central to the success of immunotherapy. PD-1/PD-L1 interactions dampen T cell activity and promote immune evasion in many cancers, including HCC. m6A modification is involved in regulating PD-L1 expression, thus influencing its interaction with immune cells. In intrahepatic cholangiocarcinoma, the demethylase ALKBH5 stabilizes PD-L1 mRNA through demethylation, maintaining its expression and suppressing T cell activation (81). Moreover, circRNAs stabilized by m6A enhance PD-L1 expression in HCC, leading to T cell dysfunction and contributing to resistance to ICIs (48, 82). These findings highlight the therapeutic potential of targeting m6A pathways to sensitize tumors to ICIs and improve immunotherapy responses.

Given the pivotal role of m6A in modulating the TIME and regulating immune checkpoints, it holds significant promise as a therapeutic target for enhancing immunotherapy. Preclinical studies have demonstrated that inhibiting METTL3 or YTHDF1 can synergize with anti-PD-1 therapy, significantly improving antitumor response and overall treatment efficacy (47, 100, 101). Furthermore, modulating m6A levels in inflammatory pathways, particularly those involving IL-6/STAT3 signaling, could reduce chronic inflammation and inhibit tumorigenesis, addressing fundamental drivers of HCC progression. This suggests that targeting m6A may not only enhance immune responses but also improve the TME, providing a more comprehensive approach for cancer treatment.

In conclusion, the intricate involvement of m6A in shaping the immune landscape and regulating immune checkpoints underscores its potential as a powerful therapeutic target in HCC. By strategically targeting m6A pathways, it may be possible to reprogram the TIME, enhance the effectiveness of ICIs, and provide novel strategies for treating HCC. Future research should focus on refining the specificity of m6A-targeting drugs, exploring their integration into combination therapies, and identifying biomarkers that can predict patient responses. These efforts could pave the way for more personalized and effective treatment options, offering new hope for patients with HCC.
